# Chopstick technique used in laparoendoscopic single site radical hysterectomy for early stage cervical cancer

**DOI:** 10.1038/s41598-021-85783-5

**Published:** 2021-03-25

**Authors:** Yanzhou Wang, Yuanyang Yao, Yuya Dou, Shuai Tang, Cheng Chen, Yudi Li, Yong Chen, Li Deng, Zhiqing Liang

**Affiliations:** Department of Obstetrics and Gynecology, Southwest Hospital Third Military Medical University, Chongqing, 400038 China

**Keywords:** Surgical oncology, Surgery, Surgical oncology

## Abstract

Laparoendoscopic single-site surgery (LESS) further minimizes the invasiveness of traditional laparoscopic surgery. However, the "chopstick" effect caused by the parallel arrangement of the instruments in the umbilicus is considered an obstacle indelicate operations. The purpose of this study was to introduce a new technique characterized by a double fulcrum formed by instruments, named the "chopstick" technique, which facilitates the expedient accomplishment of complicated surgeries such as LESS radical hysterectomy (LESS-RH). Seventy-three patients who underwent LESS-RH using the "chopstick" technique were retrospectively analyzed. The procedure was performed successfully in 72 patients. The median operative duration was 225 min, and the median intraoperative blood loss was 200 ml. Among the operations in the first 20 patients, intraoperative vascular injuries and bladder injury occurred in two patients and were repaired by LESS. Patients responded positively regarding minimal postoperative pain control. The score of satisfaction with the cosmetic outcome expressed by the patients was eight at discharge and nine 30 days later. In conclusion, this study presents the feasibility of accomplishing complicated procedures, such as radical hysterectomy, by LESS using the “chopstick” technique. This approach provides more options for both selected patients and surgeons.

## Introduction

Laparoendoscopic single-site surgery (LESS) is an optimization of laparoscopic surgery designed to further minimize the invasiveness of surgery^1^. Rather than using multiple abdominal incisionsin traditional laparoscopy, LESS is performed through a single incision positioned at the umbilicus. Studies across surgical disciplines have reported the feasibility and safety of LESS in radical prostatectomy, radical cystectomy^2,3^, and colorectal resection^4^ in patients with pelvic malignancy. Fader^5^ and Boruta^6^ described the feasibility of LESS in the treatment of various gynecological oncological conditions, including endometrial cancer, ovarian cancer, retroperitoneal pelvic lymph node dissection, and radical hysterectomy.

As one of the most complex surgeries in gynecological oncology, radical hysterectomy requires a very delicate operation. The difficulty caused by the parallel arrangement of all instruments in the umbilicus, which is called the "chopstick" effect^7^, is considered an obstaclein delicate operations. However, Asians use chopsticks every day and never experience any “difficult effect”. We found that Asians’ use of chopsticks represents an ingenious application of physics. If a single fulcrum is changed into adouble fulcrum in LESS as when Asians use chopsticks, delicate operations can be performed more efficiently. This technique with a double fulcrum was named the “chopstick" technique. Since 2016, we have explored this new technique for LESS radical hysterectomy with pelvic lymphadenectomy (LESS-RH/PLND). The objectives of this study were to evaluate the feasibility of the “chopstick" technique for use in complicated surgeries and discuss its technical points.

## Methods

A retrospective review was conducted of 72 consecutive patients who underwent LESS-RH/PLND with the “chopstick" technique from November 2016 to September 2018 at the First Affiliated Hospital of Third Military Medical University. In all cases, treatment was performed by four high-volume laparoscopic gynecologic oncology surgical specialists from a single practice. All women in this case series were 18 years or older, pathologically diagnosed with adenocarcinoma, squamous carcinoma or adenosquamous carcinoma, and classified as having International Federation of Gynecology and Obstetrics (FIGO) stage (2014) Ia1 disease with lymphovascular space invasion (LVSI) (stage Ib1 and IIa1). The study protocol was reviewed and approved by the ethics committee/institutional review board of the First Affiliated Hospital of Third Military Medical University, and all patients provided informed consent for LESS-RH/PLND after receiving thorough counseling detailing their therapeutic options. All methods were performed in accordance with relevant guidelines and regulations.

### LESS-RH with the "chopstick" technique

The "chopstick" technique consists of three parts, including the establishment of a single-site surgical platform, the arrangement of instruments, and the operative procedure.Type C1 or C2 (Querleu and Morrow's grading) RH was performed based on the preference of the surgeon. The difficult procedures of RH are ureteral unroofing and pelvic lymph node dissection. We take one of these procedures—ureteral unroofing—to illustrate how the "chopstick" technique works.

#### Establishment of a LESS platform with multiple independent channels

**Figure 1:**
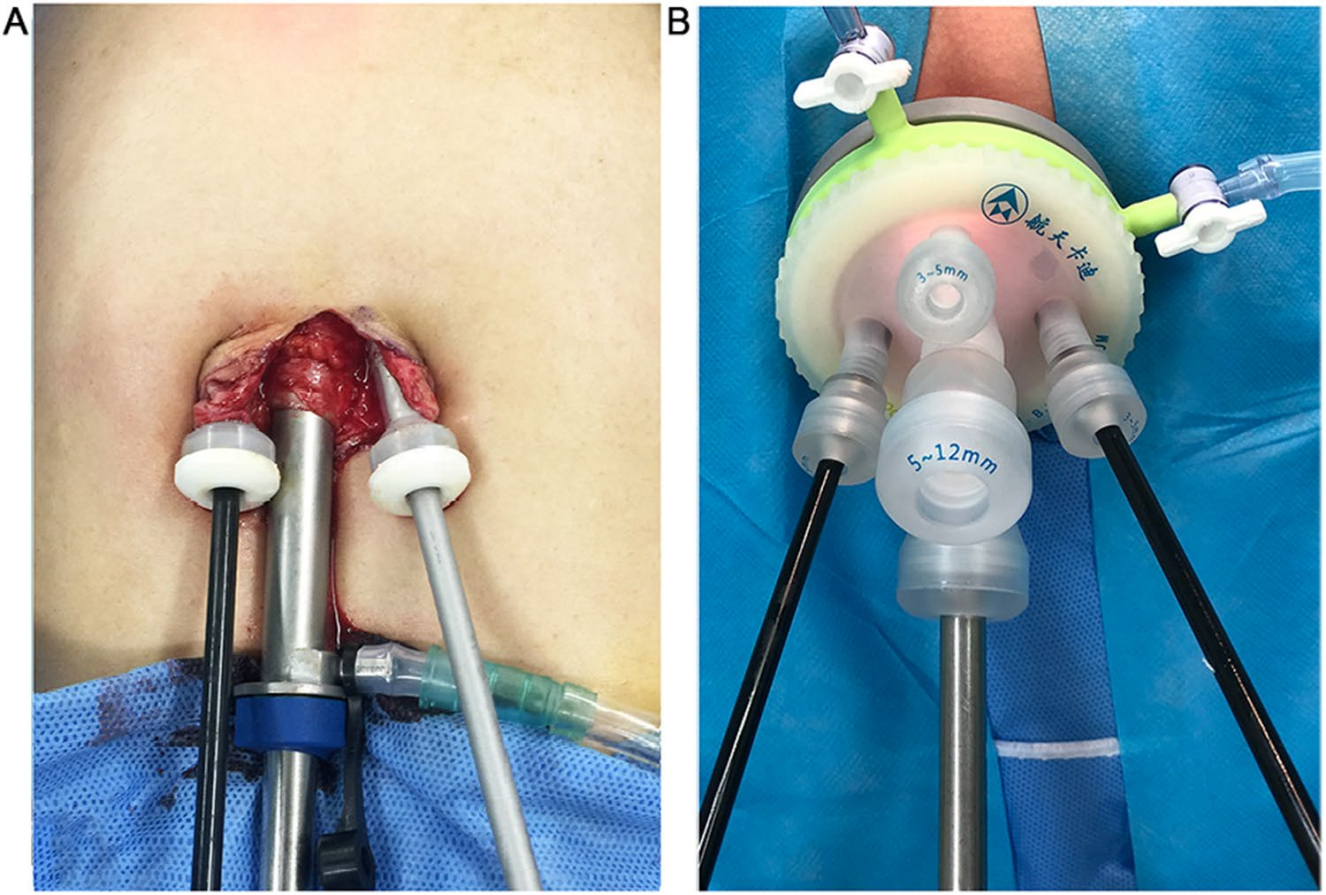
Placement of the laparoscopy platform. (**A**) Single-incision three-channel laparoscopy platform. (**B**) Multichannel-tipped single-port laparoscopy platform (HangT Port).

The principle of establishing asurgical platform for the "chopstick" technique is the formation of two or more separate channels through the abdominal wall. This can be accomplished by multiple punctures in the rectus sheath (Fig. 1A) or the use of a medium-soft, multichannel-tipped port (Fig. 1B).

#### Arrangement of the instruments for a double fulcrum

**Figure 2:**
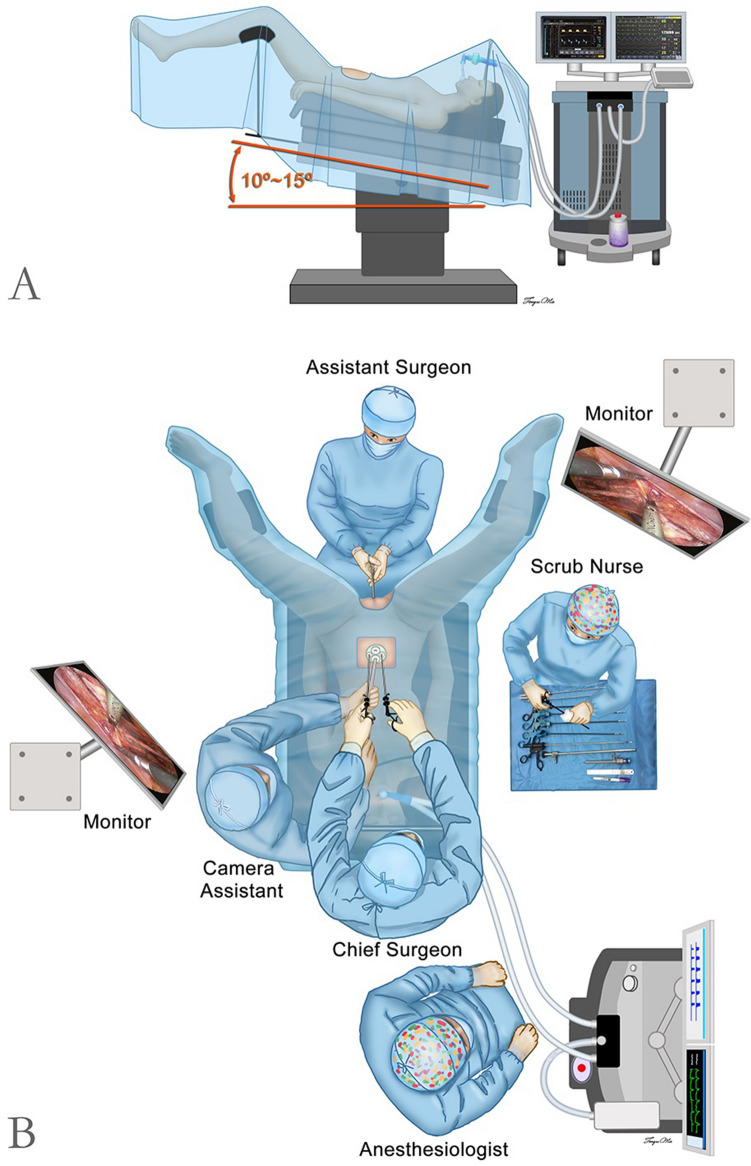
Surgical positioning and operating room setup for laparoendoscopic single-site radical hysterectomy(LESS-RH). (**A**) Surgical positioning and (**B**) operating room setup.

Two instruments were inserted into the abdominal cavity through two independent channels placed symmetrically to the left and right of the navel, and a 10-mm laparoscope was inserted at the upper part of the incision between these two channels. The two separate channels of the medium-soft port provided a double fulcrum for the forceps, and the chief surgeon stood on the cephalic side of the patient such that his arm was parallel to the forceps (Fig. 2).

#### Operative procedure for ureteral unroofing

Separation of the ureter from the cervix and vagina is a critical step of RH, which requires many delicate maneuvers. The vesicocervical space and the anterior leaf of the vesicocervical ligament were exposed by a grasper in the surgeon's left hand. Serial dissection of the vesicocervical ligament was extended to the level of ureteral insertion into the bladder by a Harmonic Scalpel in the right hand. The anterior leaf of the vesicocervical ligament is short, so the tips of the two instruments were very close to each other. The grasper in the left hand was fixed to maintain a certain tension; meanwhile, the Harmonic Scalpelin the right hand was able to move around its own fulcrum without disturbing the stability of the grasper. Thus, the roof of the ureter tunnel was opened.

### Postoperative treatment and follow-up

After surgery, patients with two or more intermediate-risk factors^8^ (LVSI, tumors > 4 cm, and deep cervical stromal invasion) were recommended to receive adjuvant radiation therapy, whereas patients with one or more high-risk factors^9^ (resection margin involvement, parametrial involvement, or lymph node involvement) were recommended to receive adjuvant concurrent chemoradiation therapy. However, some patients received adjuvant chemotherapy based on the preferences of the patient and physician. After treatment, patients were examined every three months for the first 2 years, every six months for the next 3 years, and every year thereafter.

### Outcomes

Clinical data, including patient demographics and perioperative measures, were obtained from a retrospective chart review following approval by our institutional review board. The operative duration was recorded from the first skin incision to the last suture (skin to skin). Blood loss was estimated from the contents of suction devices. The length of hospital stay was counted from the 1st postoperative day. Intraoperative complications included procedure-related organ damage and events for which intravenous medications (including blood transfusions) were needed. Postoperative complications were defined as complications occurring postoperatively up to 8 weeks after surgery^10^. Postoperative complications were graded using the Clavien-Dindo (C-D) classification of surgical complications. The cosmetic outcome of the umbilical scar was evaluated by patients on days 1 and 30 after surgery. A subjective satisfaction score ranging from 1 to 10 (with 0 being "bad" and 10 being "excellent") was assigned^11^.

### Statistical methods

SPSS 17.0 (IBM Corp, Armonk, NY, USA) was used for statistical analysis. All data were analyzed and reported as numbers (%) or medians (ranges).

## Results

A total of 73 patients were included in the study. Of these, 72 patients (98%) underwent LESS successfully, and one patient was converted to multiport laparoscopic surgery due to stage 4 endometriosis. The 72 patients’ characteristics are presented in Table 1. The median age of the patients was 47 years, and the body mass index (BMI) was 23. All the patients were of Chinese ethnicity, and 36.1% had a history of previous abdominal surgery. Surgery with a single incision with three trocars was performed in 32 patients, and the HangT port (Beijing HangTian KaDi Technology R&D Institute, Beijing, China) was used in 40 patients. The "chopstick" technique was used in all patients who underwent LESS-RH. Among them, 3 patients had stage IA1 disease combined with LVSI, one patient had stage IA2 disease, and 68 patients had stage Ib1/IIa1 disease. There were 61 patients with squamous cell carcinoma, 9 with adenocarcinoma and 2 with adenosquamous carcinoma.

The surgical outcome data are presented in Table 2. Fifteen patients were treated with type C1 LESS-RH, and 57 patients were treated with type C2 LESS-RH. Pelvic lymphadenectomy was performed in all women, and para-aortic lymphadenectomy was performed in 26(36.1%) patients. The median operative duration was 217 min, and the median intraoperative estimated blood loss (EBL) was 200(10–800) ml. Postoperative pain was evaluated at 24 h and 48 h after surgery, at which time the median visual analog pain scale score at rest was 3(0–6) and 2(0–4), respectively. The time it took to achieve a postvoid residual urine volume of less than 100 ml after removal of the urethral catheter was 14(3–100)days. The median length of postoperative hospital stay was 8(2–18) days.The median cosmetic outcome satisfaction score expressed by the patient was 8(7–10) at discharge and 9 (range, 8–10) 30 days later.

**Table 1:** Patients’ characteristics and staging (no. of surgery = 72).

Variable	n(%)	Median (range)
Age, years		47(27–67)
BMI, Kg/m^2^		23(16–31)
Parity		2(0–5)
Previous abdominal surgery, n(%)	26(36.1%)	
**FIGO clinical stage**^**a**^**, n(%)**		
IA1^b^	3(4.1%)	
IA2	1(1.3%)	
IB1	44(61.1%)	
IIA1	24(33.3%)	
**Histology, n(%)**		
Squamous	61(84.7%)	
Adenocarcinoma	9(12.5%)	
Adenosquamous	2(2.8%)	

SPSS 17.0 (IBM Corp, Armonk, NY, USA) was used for statistical analysis.The website of SPSS is https://www.ibm.com/cn-zh/analytics/spss-statistics-software.

**Table 2:** Intra- and postoperative details(no. of surgery = 72).

Variable	n(%)	Median (range)
Type C1 radical hysterectomy, n(%)	15(20.8%)	
Type C2 radical hysterectomy, n(%)	57(79.2%)	
Pelvic lymphadenectomy, n(%)	72(100%)	
Aortic lymphadenectomy, n(%)	26(36.1%)	
Operative time, min		217(115–415)
Median EBL, ml		200(10–800)
Intraoperative complication, n(%)	3(4.2%)	
Pain score (12 h)		3(0–6)
Pain score (24 h)		2(0–40)
Removal of Foley catheter, days		14(3–100)
Postoperative hospital stay, days		8(2–18)

SPSS 17.0 (IBM Corp, Armonk, NY, USA) was used for statistical analysis.The website of SPSS is https://www.ibm.com/cn-zh/analytics/spss-statistics-software.

Two intraoperative complications were observed in the first 20 patients, including 1 patient with bladder injury and concurrent left external iliac vein injury and one patient with left external iliac vein injury; these injuries were repaired by LESS with no long-term sequelae. Postoperative complications were documented in 15 of 72 (20.83%) subjects (Table 3). Thirteen patients (18.1%) had minor complications classified as C-D grade II or less, while three patients (4.2%) had complications categorized as C-D grade III. One case of ureterovaginal fistula was first diagnosed at 14 days postoperatively and was treated immediately with ureteroneocystostomy. One patient was diagnosed with paralytic ileus, and a nasogastric tube was placed to relieve abdominal distention. One case of lymphoid leakage occurred, for which a drainage tube was placed through the vagina, and the condition improved seven days later.

**Table 3:** Hospital- and patient-reported postoperative complications (no. of surgery = 72).

	Clavien-Dindo grade I–II (mild)	Clavien-Dindo grade III–IV (serious)	
	n(%)	n(%)	n(%)
Bladder dysfunction	7(9.7%)	7(9.7%)	0
Ureterovaginal fistula	1(1.4%)	0	1(1.4%)
Ileus	3(4.2%)	2(2.8%)	1(1.4%)
Wound dehiscence	1(1.4%)	1(1.4%)	0
Lymphatic fistula/lymphatic cyst	1(1.4%)	0	1(1.4%)
Deep vein thrombosis	1(1.4%)	1(1.4%)	0
Pelvic infection	2(2.8%)	2(2.8%)	0
Total	15^a^(20.8%)	13^a^ (18.1%)	3(4.2%)

SPSS 17.0 (IBM Corp, Armonk, NY, USA) was used for statistical analysis.The website of SPSS is https://www.ibm.com/cn-zh/analytics/spss-statistics-software.

^a^One patient had two complications simultaneously.

The median number of pelvic and para-aortic lymph nodes resected was 18 (10–31) and 6(4–18), respectively. Positive pelvic nodes were identified in 13 patients (18.1%). Pathological examination of the vaginal margin status yielded negative findings. Positive parametrium was found in 1 patient, deep stromal invasion in 37 patients, and LVSI in 13 patients. Twenty-eight patients were treated with postoperative concurrent chemoradiotherapy, 11 patients with intermediate-risk factors were treated with radiotherapy, and three patients were treated with chemotherapy postoperatively (Table 4).

**Table 4:** Histological risk factors of the patients and the adjuvant therapy received (no. of surgery = 72).

Variable	n(%)	Median (range)
Tumor size, mm		25(0–40)
Pelvic lymph nodes		18(10–31)
Para-aortic lymph nodes		6(4–18)
Lymph node positivity, n(%)	13(18.1%)	
Lymphovascular space invasion, n(%)	13(18.0%)	
Deep stromal invasion, n(%)	37(51.4%)	
Parametrial width, cm		2(1–4)
Positive parametrial margins, n(%)	1(1.4%)	
Positive vaginal margins, n(%)	0(0%)	
**Adjuvant treatment, n(%)**		
Radiotherapy	11(15.2%)	
Concurrent chemoradiotherapy	28(38.8%)	
Chemotherapy	3(4.1%)	
Recurrence, n(%)	5(6.9%)	
**Site of recurrence**^**a**^**, n(%)**		
Local	1(1.4%)	
Regional	2(2.8)	
Distant	3(4.1%)	
Death of disease, n(%)	1(1.4%)	

SPSS 17.0 (IBM Corp, Armonk, NY, USA) was used for statistical analysis.The website of SPSS is https://www.ibm.com/cn-zh/analytics/spss-statistics-software.

^a^One patient had multiple sites of recurrence.

After a median follow-up duration of 29 months(16–39), five patients had recurrence. The anatomical site of recurrence was the vaginal stump in one patient, the pelvis in one patient, the pelvic and para-aortic lymph nodes in one patient, and the lung in 2 patients.

There was one case of mortality. The patient had FIGO stage IIa1 squamous cell carcinoma with no intermediate-risk factors or high-risk factors.Therefore, adjuvant therapy was not administered to this patient; however, pelvic recurrence was found six months after surgery. She received adjuvant chemoradiotherapy and died 15 months after recurrence.

## Discussion

This report demonstrates the feasibility of the "chopstick" technique in operations as complicated as LESS-RH/PLND and representsthe largest public experience in the treatment of early cervical cancer by LESS. In the majority (98%) of these 72 patients, we were able to complete the surgery without the need for conversion to multiport laparoscopy, which is comparable to recently published data for the conversion of multiport laparoscopy to laparotomy^12^. The median number of pelvic and para-aortic lymph nodes resected and the pathological results of the parametrium and the vaginal margin were the same as those of routine clinical practice.

**Figure 3:**
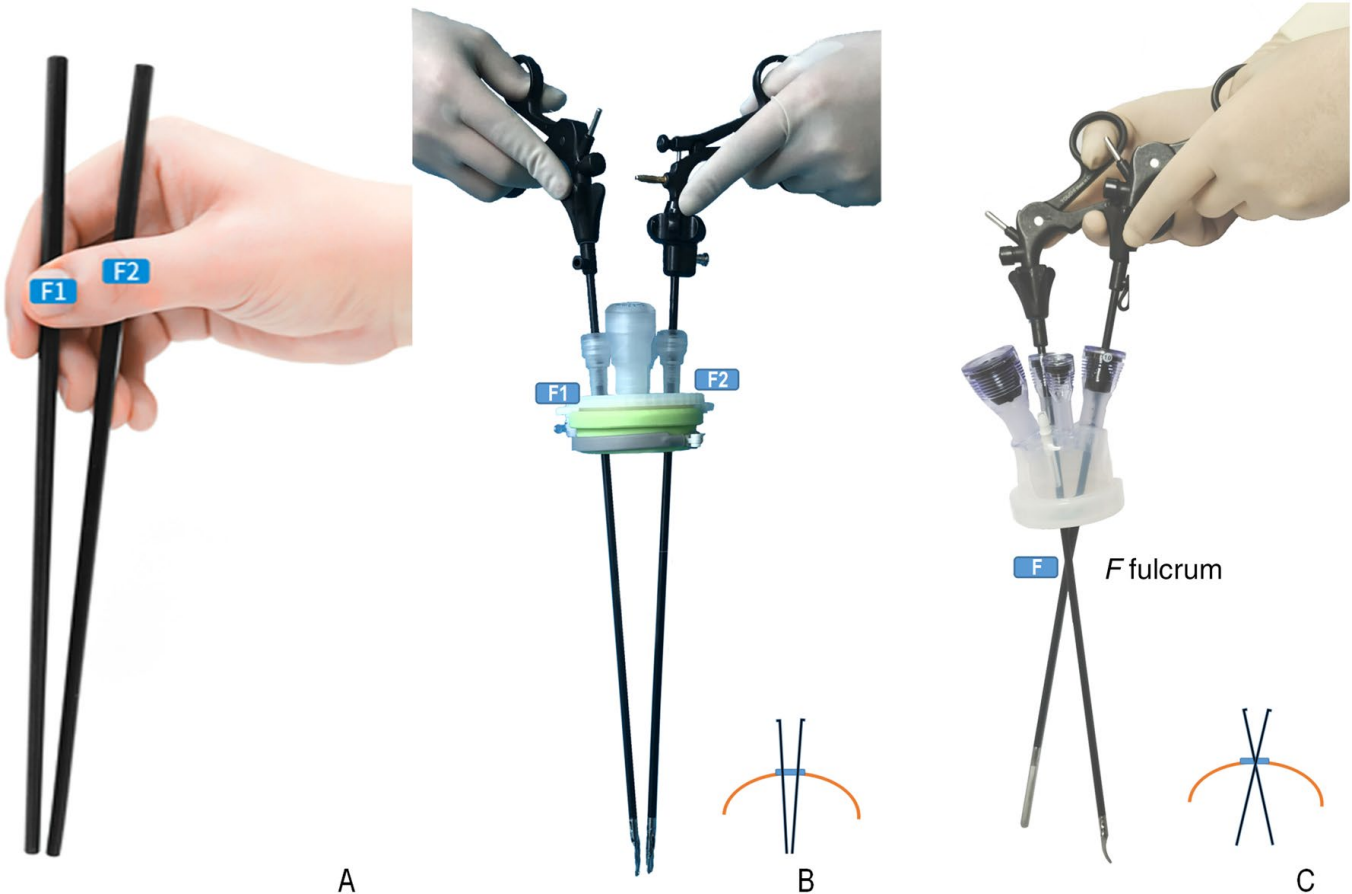
Instrument arrangement in the "chopstick" technique and the "cross" technique. (**A**) Correct method for holding chopsticks. (**B**) The "chopstick" technique with double fulcrum. (**C**) The "cross" technique with single fulcrum formed by two instruments crossing.

The completion of radical hysterectomy by LESS at our institution depends on the emergence of the “chopstick” technique and its continuous development. The arrangement of forceps in LESS has been compared to that of chopsticks in many studies in the literature. Moreover, the arrangement caused the lack of a surgical triangle and increased the difficulty of the operation, which was referred to as the chopstick effect by some surgeons^7,13^. The technique introduced in our study is based on this kind of chopstick-like arrangement. More importantly, the movement of forceps during the operation is based on the same physical principle as the use of chopsticks. The use of chopsticks varies from person to person but can be divided into two categories according to physical principles: the “parallel method” and the “cross method”. It is generally believed that the parallel method is correct. The key of this method is that the hand-held points of the two chopsticks are separated from each other; regardless of which fingers are used to control the chopsticks, the two chopsticks have their own independent fulcrum and do not interfere with each other (Figure 3A). Similarly, there are two kinds of techniques for using forceps in single-port laparoscopy, including the cross technique^14^ and the “chopstick” technique we introduced. There are different principles for movement of the forceps in the two techniques. In the cross technique, the forceps are moved using a single fulcrum, while in the “chopstick” technique, the forceps are moved using a double fulcrum, similar to chopsticks when correctly used.

Two considerations for obtaining the correct “chopstick technique” were necessary, including establishing a channel with a double fulcrum and changing the position of the surgeon. In single-incision three-channel laparoscopy, the double fulcrum is created by two incisions in the rectus sheath. When using the HangT port, the fulcrum is the port's channel, whose distance is fixed as designed by the manufacturer (Fig. 3B). To allow the surgeon's arm and the instrument to become parallel and form the chopsticks that move around the fulcrum to perform pelvic surgery, the surgeon needs to stand on the patient's cephalic side.

On account of the challenges of LESS, there has been extensive development of instruments and surgical techniques. Many specialized instruments, including a flexible-tip laparoscope and some articulating or prebent instruments, have been invented to provide more space for a surgical triangle. However, their actual efficacy needs further verification. Robotic systems, such as the da Vinci Si Surgical System, also require space in the surgical field to provide manipulators with high degrees of freedom^13^. However, the efficiency of both EndoWrist movements and semirigid manipulators has been criticized, and the drawbacks of this new technology in LESS have been noted. Several surgical techniques have been described in the literature to solve the mutual influence of two hands due to adjacent instruments. In the glove-port technique^15^ and the cross-handed technique^14^, the instruments are arranged specifically, shown as the right instrument on the left side of the target and the left instrument on the right, which is known as the "cross" technique (Fig. 3C). The two instruments enter the abdominal cavity through the umbilical single-port access device, forming a cross in the abdominal wall, which is the instrument's fulcrum. Although these techniques can be used to perform most basic gynecological operations, the instruments may interfere with each other because of their joint fulcrum. It is generally agreed in LESS that the farther apart the two instrument tips are, the more convenient the operation is to avoid collisions between the two hands. However, when a delicate maneuver is performed in laparoscopic surgery for gynecological cancers, especially in dissection of the vesicocervical ligament, the tips of the instruments need to be very close. Thus, the “chopstick”technique may havea more significant advantage inthis context because of its two independent fulcrums.

The proportion of women successfully treated by the intended approach without conversion to another approach is often used as a primary outcome to evaluate a new operation. Regarding our results, the conversion rate was 2%, which is lower than that reported by Boruta in their early experience with LESS-RH, wherein the procedure was converted to laparotomy in 2 of 22 women^16^. With any new surgical technique, an assessment of the operative duration and EBL is essential in determining feasibility and safety. The operative duration (251 min) and EBL (200 ml) were not significantly affected by the proposed technique. Moreover, complications did not increase, with a rate of C-D grade III-IV complications of 4.2%. The three cases of severe intraoperative complications consisting of bladder injury and external iliac injury occurred in the first 20 patients treated by different surgeons, which indicates that individual surgeons need to go through alearning curve to master the new technique. All patients were delighted with the cosmetic results and postoperative pain control, which may be advantages of LESS. No cancer cells were observed at the outer edge of the tissue that was removed in our LESS series. The median number of lymph nodes retrieved was similar to that reported for multiport laparoscopy or laparotomy, further supporting the feasibility of LESS^11,17,18^.

We cannot deny that the recurrence rate in this study is higher than that in the laparoscopic approach to cervical cancer (LACC) study, but there is not much difference compared with the rates in previous retrospective studies. There are many influential factors of the oncological outcome, and the effect of the surgical approach and technique needs further research. After the LACC trial was published in the New England Journal of Medicine, the minimally invasive treatment of cervical cancer became controversial. Patients were to be carefully counseled if minimally invasive surgery was offered. However, a complete abandonment of minimally invasive surgery would seem excessive. The purpose of our study is only to show that the "chopstick" technique is feasible to perform and is one of the available minimally invasive treatment options in surgery for gynecological tumors.

### Limitations

Although 72 RH procedures were successfully performed with this technique, there are still limitations. First, the surgeon is far away from the operative field, which leads to a great deal of strain on the surgeon’s back. In addition, the chief surgeon's position may occupy the anesthesiologist's territory and carry the risk of compressing the tracheal tube. At present, this technique has only been applied at our institution. There are only observational data, and no comparative studies with other techniques have been performed; hence, the feasibility and safety of the technique have not been fully affirmed.

## Conclusion

In this study, we used the "chopstick" technique to complete LESS-RH/PLND in 72 patients with early cervical cancer, confirming the feasibility of the "chopstick" technique in complex surgery for gynecological tumors. The advantages of the "chopstick" technique are the double fulcrum, usage of traditional instruments, and bimanual operation. However, this was a single-center study of LESS. More extensive clinical research is needed to confirm the universal value of the "chopstick" technique.
